# Long noncoding RNA ARHGAP27P1 inhibits gastric cancer cell proliferation and cell cycle progression through epigenetically regulating p15 and p16

**DOI:** 10.18632/aging.102377

**Published:** 2019-10-30

**Authors:** Guohua Zhang, Ying Xu, Chen Zou, Yinbing Tang, Jiawei Lu, Zhigang Gong, Gui Ma, Wenbo Zhang, Pengcheng Jiang

**Affiliations:** 1 Department of General Surgery, Affiliated People’s Hospital of Jiangsu University, Zhenjiang, China; 2 Department of General Surgery, Peony People’s Hospital, Heze, China; 3 Department of Laboratory Center, Affiliated People’s Hospital of Jiangsu University, Zhenjiang, China

**Keywords:** ARHGAP27P1, JMJD3, p15, p16, gastric cancer (GC)

## Abstract

Long noncoding RNAs (lncRNAs) have emerged as important regulators in the development and progression of gastric cancer (GC). ARHGAP27P1 is a pseudogene-derived lncRNA, and it has been found to be associated with GC in our preliminary study, but this association has not been studied further. Herein, we confirmed that ARHGAP27P1 was significantly downregulated in GC tissues, plasma and cells. Low expression of ARHGAP27P1 was closely associated with advanced TNM stage, increased invasion depth and lymphatic metastasis. Low ARHGAP27P1 expression also predicted a poor prognosis in GC patients. Functionally, overexpression of ARHGAP27P1 inhibited proliferation, invasion, and migration in GC cells, while silencing of ARHGAP27P1 showed the opposite effects. Mechanistic investigations showed that ARHGAP27P1 had a key role in G0/G1 arrest. We further demonstrated that ARHGAP27P1 was associated with Jumonji-domain containing 3 (JMJD3) and that this association was required for the demethylation of H3K27me3, thereby epigenetically activating expression of p15, p16 and p57. Moreover, knockdown of JMJD3, p15, or p16 consistently reversed the inhibitory effects of ARHGAP27P1 in cell proliferation and cell cycle progression. Taken together, these results suggest that lncRNA ARHGAP27P1, as a novel cell cycle regulator, may serve as a potential target for GC prevention and treatment in human GC.

## INTRODUCTION

Gastric cancer (GC) is the fifth most frequently diagnosed cancer and the third leading cause of cancer death worldwide [1]. Despite efforts to improve diagnostic techniques and patient management, the 5-year overall survival (OS) rate for patients with GC remains unsatisfactory because lots of patients are diagnosed at an advanced stage accompanied by lymphatic metastasis [2, 3]. Thus, a better understanding of the mechanisms underlying GC occurrence and progression is essential for improving diagnosis and treatment of the disease.

Recent advances in whole-genome sequencing technology have led to the discovery of a new class of regulatory RNAs, i.e. long noncoding RNAs (lncRNAs), which are defined as transcribed RNA molecules longer than 200 nucleotides and lacking protein-coding capacity [4, 5]. Emerging evidence suggests that lncRNAs may play critical roles in cellular development, differentiation, and many other biological processes [4–7]. The aberrant expression of lncRNAs has also been shown in various malignancies including GC [8–10]. In addition, lncRNAs may mediate oncogenic or oncosuppressive effects and may be cancer biomarkers and therapeutic targets [9]. Molecular mechanisms of lncRNAs are diverse. They have been shown to regulate gene expression by executing as signals, decoys, guides and scaffolds [5, 8, 10]. For example, metastasis-associated lung adenocarcinoma transcript 1 (MALAT1) functions as a competing endogenous RNA (ceRNA) to attenuate the inhibitory effect of miR-23b-3p on autophagy-related protein 12 (ATG12), and then leads to chemo-induced autophagy and chemoresistance in GC [11]. In contrast, lncRNA GAS5 enhances G1 cell cycle arrest via binding to YBX1 to regulate p21 expression in GC [12].

Of the diverse array of putative molecular and biological functions assigned to lncRNAs, one attractive perspective in epigenetic research has been the hypothesis that lncRNAs directly interact with the proteins involved in the modulation of chromatin conformation. Epigenetic modifiers are among the most frequent protein partners of lncRNAs that have been identified to date, of which histone methylransferases and protein members of the polycomb repressive complex 2 (PRC2) have received considerable attention [13]. Indeed, it has been reported that approximately 20% of all human lncRNAs act to physically associate with PRC2, suggesting that lncRNAs may have a general role in recruiting PRC2 to their target genes [14]. We have also previously reported that lncRNA SNHG17 acts to interact with PRC2, thereby promoting cell cycle progression and proliferation of GC cells by epigenetic silencing of the major cyclin-dependent kinase inhibitors (CKIs) including p15 and p57 [15]. Other epigenetic modifiers including LSD1, SMYD3, WDR5, KMT2C, SET1C, ING2 and PAQR3 may also function as potential partners for lncRNA [16–18]. For example, the interaction of lncRNA EZR-AS1 with SMYD3 has been shown to enhance SMYD3-dependent histone H3 lysine 4 (H3K4) methylation and activate EZR transcription in esophageal squamous cell carcinoma (ESCC) [17]. Besides, lncRNA HOXD-AS1 recruits WDR5 to directly regulate the expression of target genes by mediating H3K4 tri-methylation [18]. Though a small portion of lncRNAs have been functionally characterized in epigenetic modulation, many members in this class remain uncharacterized and associated research is still urgently needed.

The Rho GTPase activating protein (ARHGAP) family is a class of Rho homologous GTPase activating proteins that cause tumor formation through the dysregulation of Rho/Rac/Cdc42-like GTPases [19]. Emerging evidence has demonstrated crucial roles of ARHGAP family in the tumorigenesis and progression of diverse cancers. For example, ARHGAP1 is a factor comprising GTPase-activating protein, which enhances intrinsic GTPase activity, leading to G protein inactivation. ARHGAP1 has been shown to regulate the epithelial-to-mesenchymal transition (EMT) by inhibiting RhoA/ROCK signaling [20]. ARHGAP20 gene is located within the 11q23.1 commonly deleted region of breast cancer [21]. Besides, ARHGAP8 acts to promote the development of colorectal cancer (CRC) by encoding a novel RHOGAP with a unique functional domain [22]. ARHGAP30 induces apoptosis of CRC cells by regulating the expression of p300 to promote p53 acetylation and functional activation [23]. Located within chromosome 17q24.1, ARHGAP27 pseudogene 1 (ARHGAP27P1) is a pseudogene-derived lncRNA with a length of 2891 nt. In general, pseudogenes are dysfunctional copies of protein-coding genes, representing an intriguing class of lncRNAs. Traditionally considered as “genomic junk”, recent researches have pointed towards a functional role of many pseudogenes in tumorigenesis or tumor suppression. For example, PTEN pseudogene 1 (PTENP1) may downregulate the expression of PTEN by recruiting DNA methyltransferase 3a, thereby inhibiting apoptosis of GC cells [24]. Using RNA-sequencing data from two cohorts of lung adenocarcinoma patients, Stewart GL et al. discovered 104 deregulated pseudogene-derived lncRNAs, many of which are expressed from the loci of pseudogenes related to known cancer genes [25]. Their findings further suggest the lncRNA-pseudogene-protein-coding gene axis as a prominent mechanism of cancer gene regulation in lung adenocarcinoma. Besides executing as a synergistic or antagonistic cofactor of its parental genes, many other mechanisms including epigenetic regulation, alternative splicing and RNA decoy have also been reported for pseudogene-derived lncRNAs in the oncogenesis of cancers [26–28].

Here, we report, for the first time, the expression pattern, function and regulatory mechanism of ARHGAP27P1 in GC. The results demonstrated that ARHGAP27P1 expression was significantly decreased in GC tissue samples and cell lines. Low ARHGAP27P1 expression was significantly correlated with advanced TNM stage and independently predicted a poor OS of GC patients. In addition, we investigated the effects of ARHGAP27P1 expression on GC cell phenotype *in vitro* and *in vivo* with the gain- and loss-of-function studies. Moreover, we also showed that ARHGAP27P1 could bind to Jumonji-domain containing 3 (JMJD3) to activate p15, p16 and p57 transcription. Consistently, silencing of JMJD3, p15, or p16 reversed the inhibitory effects of ARHGAP27P1 in cell proliferation and cell cycle progression. Therefore, this study suggests a tumor suppressor role of ARHGAP27P1 and expands our understanding of the role of lncRNAs as an epigenetic regulator in GC oncogenesis and progression.

## RESULTS

### Downregulation of ARHGAP27P1 was associated with advanced disease and predicted poorer prognosis

**Figure 1:**
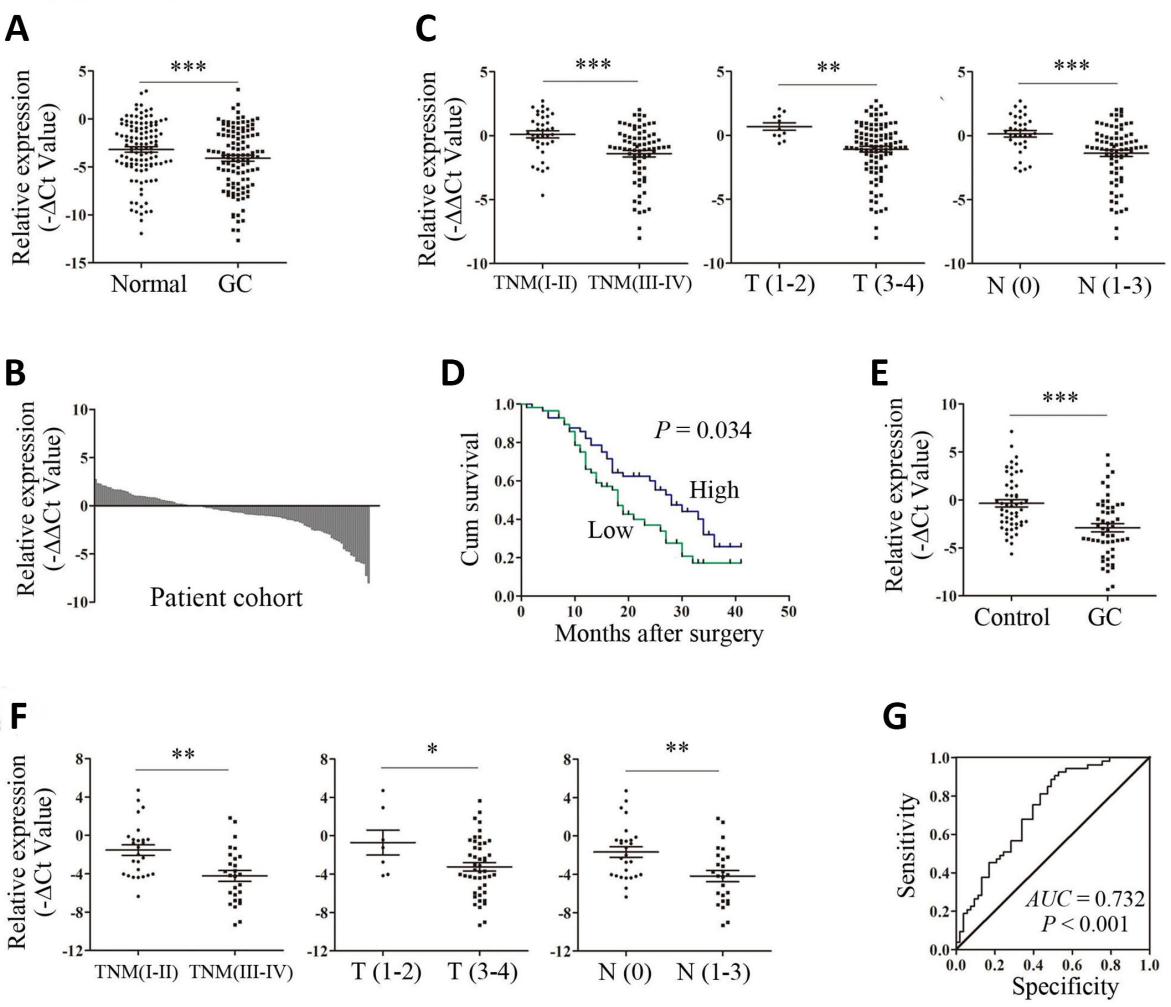
**Upregulation of ARHGAP27P1 predicted advanced disease and poorer OS of GC.** (**A**) The ARHGAP27P1 levels in GC tissues and adjacent noncancerous tissues were detected by RT-qPCR (n=112). (**B**) The fold change of ARHGAP27P1 expression in GC tissues compared with that in the paired noncancerous tissues for each patient. (**C**) The correlation between ARHGAP27P1 expression and TNM stage, invasion depth and lymphatic metastasis. (**D**) Kaplan-Meier analysis of OS according to ARHGAP27P1 expression levels. (**E**) The expression levels of plasma ARHGAP27P1 in GC patients (n=53) and healthy controls (n=53) were detected by RT-qPCR. (**F**) The correlation between plasma ARHGAP27P1 expression and TNM stage, invasion depth and lymphatic metastasis. (**G**) The ROC curve of plasma ARHGAP27P1 for diagnosis of GC. *P < 0.05, **P < 0.01, ***P < 0.001.

To explore the expression profile of ARHGAP27P1 in GC, we first detected ARHGAP27P1 expression levels in a cohort of 112 paired GC and adjacent noncancerous tissues by RT-qPCR. As shown in Figure 1A, the expression levels of ARHGAP27P1 were lower in tumor tissues. In addition, the expression of ARHGAP27P1 was downregulated in 64.3% (72/112) of GC tissues compared with that in the adjacent normal tissues (Figure 1B). Given that the differences between groups were not large, these results were still worth further investigation. To further explore the relationship between ARHGAP27P1 levels and clinicopathological parameters in GC patients, the patients were divided into high (n = 56, fold change ≥ median) and low (n = 56, fold change < median) expression groups according to the median value of ARHGAP27P1 levels. As shown in Table 1 and Figure 1C, lower ARHGAP27P1 levels were correlated with advanced TNM stage, increased invasion depth and lymphatic metastasis.

**Table 1:** Correlation between ARHGAP27P1 expression and patients’ clinicopathological parameters.

**Features**	**Number**	**ARHGAP27P1**		***P* value**
		**High n=56**	**Low n=56**	
Gender				
Male	87	40	47	0.112
Female	25	16	9	
Age				
< 60	23	11	12	0.815
≥ 60	89	45	44	
Drink				
No	83	45	38	0.131
Yes	29	11	18	
Smoke				
No	79	40	39	0.836
Yes	33	16	17	
Location				
Proximal	63	30	33	0.788
Middle	22	11	11	
Distal	27	15	12	
Differentiation				
Moderate	47	25	22	0.566
Poor	65	31	34	
Venous or				
perineural invasion				
Negative	70	37	33	0.435
Positive	42	19	23	
Tumor size				
≤ 5cm	72	39	33	0.237
>5cm	40	17	23	
TNM stage				
I-II	37	27	10	0.001
III-IV	75	29	46	
T stage				
T1-2	11	10	1	0.004
T3-4	101	46	55	
N stage				
N0	34	25	9	0.001
N1-3	78	31	47	
M stage				
M0	110	55	55	1.000
M1	2	1	1	

No relationship between ARHGAP27P1 expression and other factors, for example, gender, age, drink, smoke, tumor location, tumor differentiation, tumor size or distant metastasis, was found in our study (Table 1). To further evaluate the value of ARHGAP27P1 in the prognosis of GC, we performed Kaplan-Meier survival analysis and log-rank tests. The results showed that downregulation of ARHGAP27P1 predicted a poorer OS in patients with GC (Figure 1D). Univariate and multivariate Cox regression analyses revealed that ARHGAP27P1 expression was an independent prognostic factor for OS (P = 0.039 and 0.048, respectively) in GC patients (Table 2). Thus, these data identified that low ARHGAP27P1 expression acted as an indicator for poor survival of GC patients.

**Table 2:** Univariate and multivariate analyses of overall survival in patients with GC.

**Features**	**Univariate analysis**		**Multivariate analysis**	
	**HR (95% CI)**	***P* value**	**HR (95% CI)**	***P* value**
Gender	1.068 (0.610–1.868)	0.819	-	-
Age	0.857 (0.500–1.468)	0.573	-	-
Drink	1.153 (0.674–1.974)	0.603	-	-
Smoke	0.835 (0.492–1.418)	0.505	-	-
Differentiation	0.902 (0.556–1.463)	0.675	-	-
Tumor location	1.394 (0.870–2.235)	0.168	-	-
Tumor size	1.183 (0.725–1.931)	0.501	-	-
TNM stage	1.655 (1.013–2.704)	0.044	-	-
T stage	1.774 (0.714–4.412)	0.217	-	-
N stage	1.675 (0.981–2.863)	0.059	-	-
M stage	12.477 (2.766–56.276)	0.001	10.538 (2.361–47.029)	0.002
ARHGAP27P1 expression	0.608 (0.379–0.974)	0.039	0.620 (0.386–0.996)	0.048

### Plasma ARHGAP27P1 demonstrated moderate accuracy in diagnosis of GC

To further explore the potential utility of ARHGAP27P1 for GC diagnosis, we detected the expression of ARHGAP27P1 in plasma samples from 53 randomly selected GC patients and paired healthy donors. As shown in Figure 1E, the expression levels of plasma ARHGAP27P1 were significantly lower in GC patients than those in healthy controls. Consistently, downregulation of plasma ARHGAP27P1 was significantly associated with advanced TNM stage, increased invasion depth and lymphatic metastasis, respectively (Figure 1F). We then plotted ROC curve to assess the diagnostic value of plasma ARHGAP27P1 for GC. As demonstrated in Figure 1G, the AUC was 0.732 (95% CI, 0.636–0.827, *P* < 0.001), with the diagnostic sensitivity and specificity measuring 75.5 and 60.4% with the cut-off value of −2.321, respectively. These results indicated that plasma ARHGAP27P1 could be a potential predictor for GC diagnosis.

### ARHGAP27P1 inhibited cell proliferation, cell cycle progression, migration and invasion *in vitro*

**Figure 2:**
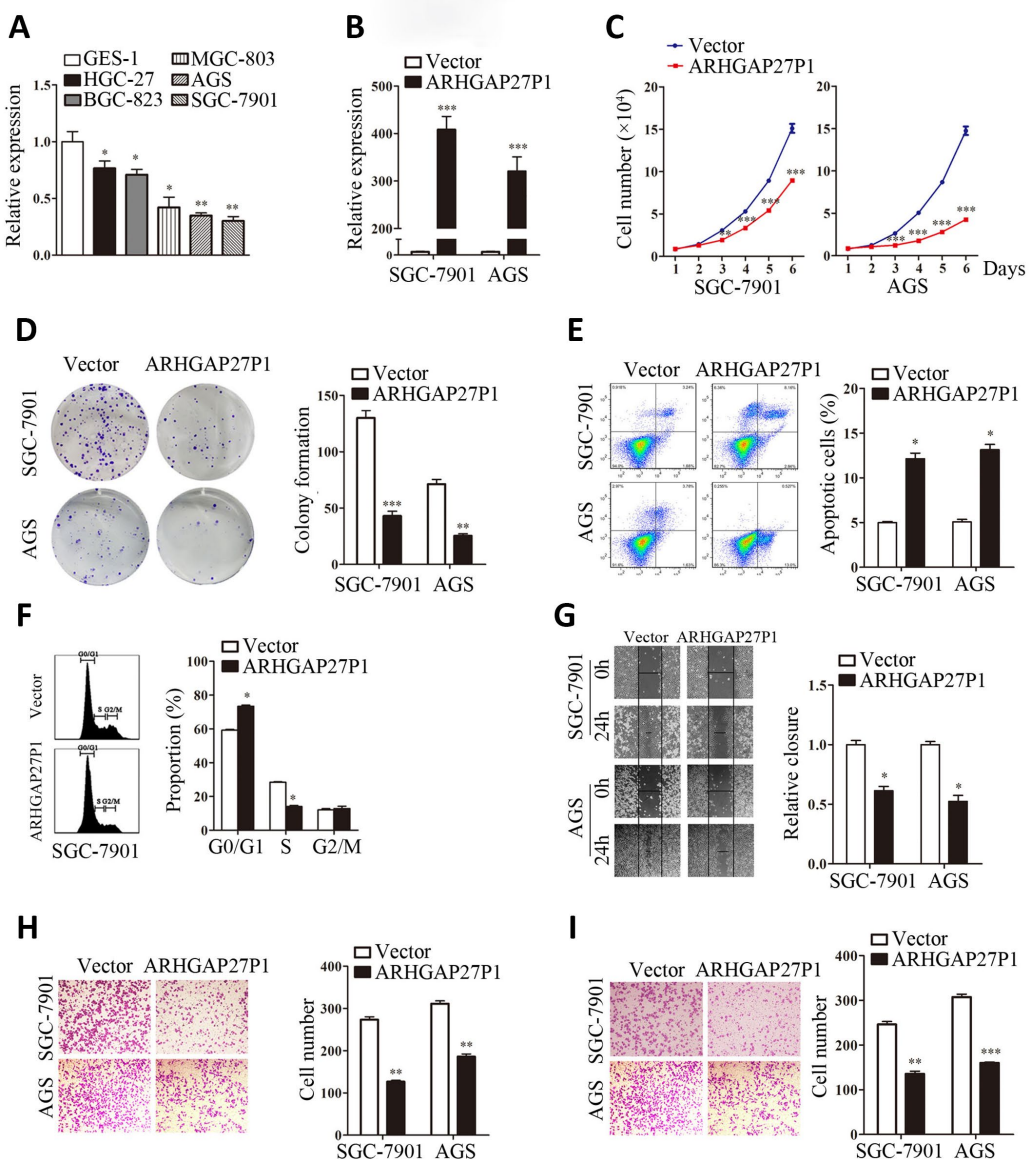
**Overexpression of ARHGAP27P1 inhibited proliferation, cell cycle progression, invasion and migration, while induced apoptosis of GC cells.** (**A**) RT-qPCR analysis of ARHGAP27P1 expression in GES-1 cells and different GC cells. (**B**) Relative ARHGAP27P1 levels in SGC-7901 and AGS cells transfected with pcDNA or pcDNA-ARHGAP27P1. (**C**) Cell proliferation in SGC-7901 and AGS cells transfected with pcDNA or pcDNA-ARHGAP27P1 was determined by cell counting assays. (**D**) Colony formation assays. (**E**) Cell apoptosis assays. (**F**) Cell cycle assays. (**G**) Wound-healing assays. (**H**) Transwell migration assays. (**I**) Transwell invasion assays. *P < 0.05, **P < 0.01, ***P < 0.001 versus GES-1 or vector.

**Figure 3:**
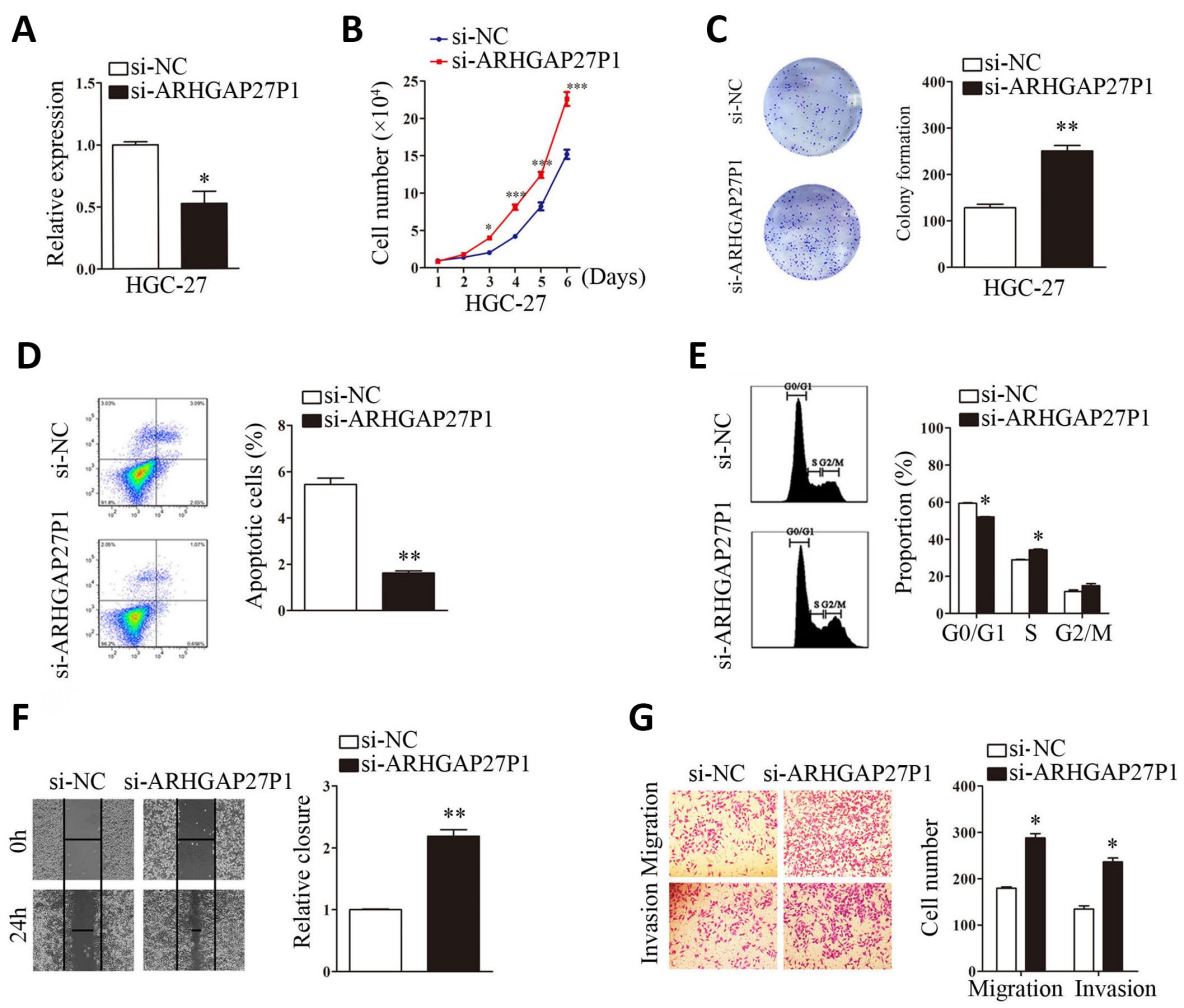
**Silencing of ARHGAP27P1 promoted proliferation, cell cycle progression, invasion and migration, and inhibited apoptosis of HGC-27 cells.** Scramble siRNA (si-NC) or ARHGAP27P1 siRNA (si-ARHGAP27P1) was transfected into HGC-27 cells. (**A**) Knockdown efficiency of si-ARHGAP27P1 was determined by RT-qPCR. (**B**) Cell proliferation was determined by cell counting assays in ARHGAP27P1-silencing HGC-27 cells. (**C**) Colony formation assays. (**D**) Cell apoptosis assays. (**E**) Cell cycle assays. (**F**) Wound healing assays. (**G**) Transwell migration and invasion assays. *P < 0.05, **P < 0.01 versus si-NC.

To gain insight into functional role of ARHGAP27P1 in GC, we first determined the expression of ARHGAP27P1 in diverse GC cell lines (MGC-803, AGS, HGC-27, BGC-823 and SGC-7901). Our data showed that ARHGAP27P1 was generally downregulated in the GC cells compared with that in GES-1 (Figure 2A). The relative low expression cell lines (SGC-7901 and AGS) were then selected as target cells for overexpression experiments, whereas the relative high expression cell line (HGC-27) were selected for knockdown experiments. The effectiveness of overexpression or knockdown was shown in Figure 2B, 3A. Cell counting assays showed that overexpression of ARHGAP27P1 led to growth retardation of SGC-7901 and AGS cells compared with the control cells (Figure 2C). Consistently, colony formation assays showed that the clonogenic survival was obviously decreased following ARHGAP27P1 overexpression. (Figure 2D). Next, flow cytometric analysis was performed to examine whether ARHGAP27P1 affected the proliferation of GC cells by altering cell cycle progression or apoptosis. The results revealed that ARHGAP27P1 overexpression induced apoptosis and cell cycle arrest at the G0/G1 phase (Figure 2E, 2F). Cell motility was further measured by wound-healing assays and transwell assays. As shown in Figure 2G–2I, ARHGAP27P1 overexpression impeded the capacity of wound closure, migration and invasion of SGC-7901 and AGS cells. Conversely, as opposed to the effects of ARHGAP27P1 overexpression, silencing of ARHGAP27P1 promoted the proliferation, cell cycle progression and cell motility of HGC-27 cells (Figure 3B–3G). Collectively, these findings suggested that ARHGAP27P1 rendered a less aggressive phenotype of GC cells.

### ARHGAP27P1 promoted expression of p15, p16 and p57

**Figure 4:**
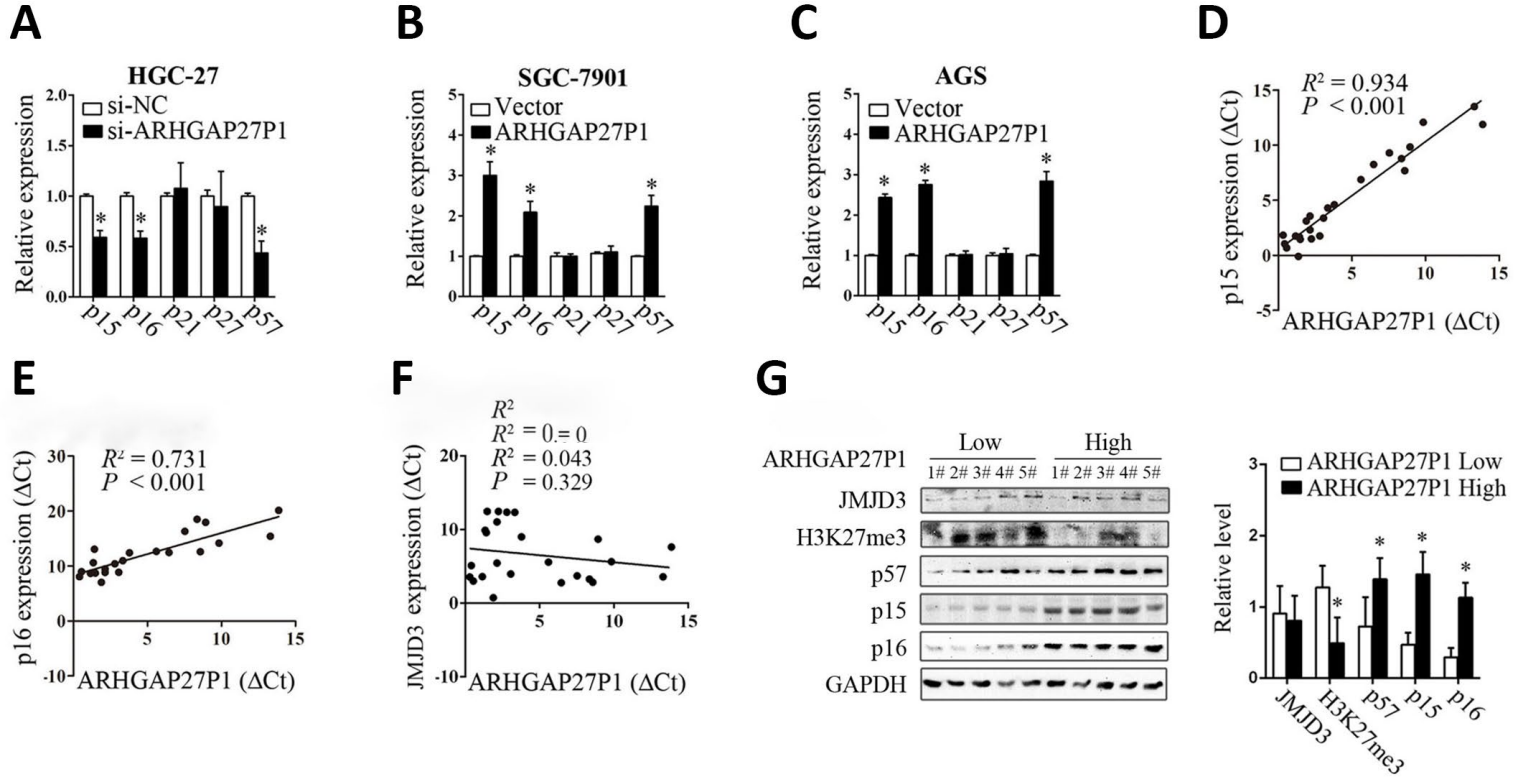
**ARHGAP27P1 affected the expression of p15, p16 and p57.** (**A**) RT-qPCR analysis of the main CKIs including p15, p16, p21, p27 and p57 in HGC-27 cells transfected with si-ARHGAP27P1 or si-NC. (**B** and **C**) RT-qPCR analysis of the main CKIs in SGC-7901 or AGS transfected with pcDNA-ARHGAP27P1 or pcDNA. (**D**–**F**) Spearman’s correlation analysis of the relationship between the expression of ARHGAP27P1 and that of p15, p16 or JMJD3 in 24 GC tissues. (**G**) Western blot analysis of JMJD3, H3K27me3, p15, p16 and p57 in 5 cases of cancerous tissues with high ARHGAP27P1 expression and 5 cases with low ARHGAP27P1 expression. *P < 0.05 versus si-NC, vector or low ARHGAP27P1 expression.

To investigate the possible mechanisms by which ARHGAP27P1 regulates the biological phenotypes of GC cells, we screened the key genes associated with cell proliferation, apoptosis, cell cycle progression, angiogenesis, EMT and microenvironment remodeling. Consistent with the role of ARHGAP27P1 in cell cycle modulation, RT-qPCR and Western blot results showed that ARHGAP27P1 mainly affected the expression of CKIs including p15, p16 and p57, but not the expression of the other potential targets (Figure 4A–4C and Supplementary Figure 1A–1C). These results suggested that ARHGAP27P1 might contribute to cell cycle arrest through regulating expression of the tumor suppressor p15, p16 and p57. We then performed Spearman’s correlation analysis to assess the relationship between the expression of ARHGAP27P1 and that of p15 or p16 in 24 cases of GC tissues, respectively. As shown in Figure 4D and 4E, a positive correlation between the expression of ARHGAP27P1 and that of p15 or p16 was also observed. In addition, we also detected the protein levels of p15, p16 and p57 in 5 cases of cancerous tissues with high ARHGAP27P1 expression and 5 cases with low ARHGAP27P1 expression. The results consistently showed that the ARHGAP27P1 expression inversely associated with the protein expression of p15, p16 and p57 (Figure 4G). Therefore, these data suggested that ARHGAP27P1 induced cell cycle arrest through regulating p15, p16 and p57.

### ARHGAP27P1 epigenetically activated p15, p16 and p57 transcription by binding to JMJD3

**Figure 5:**
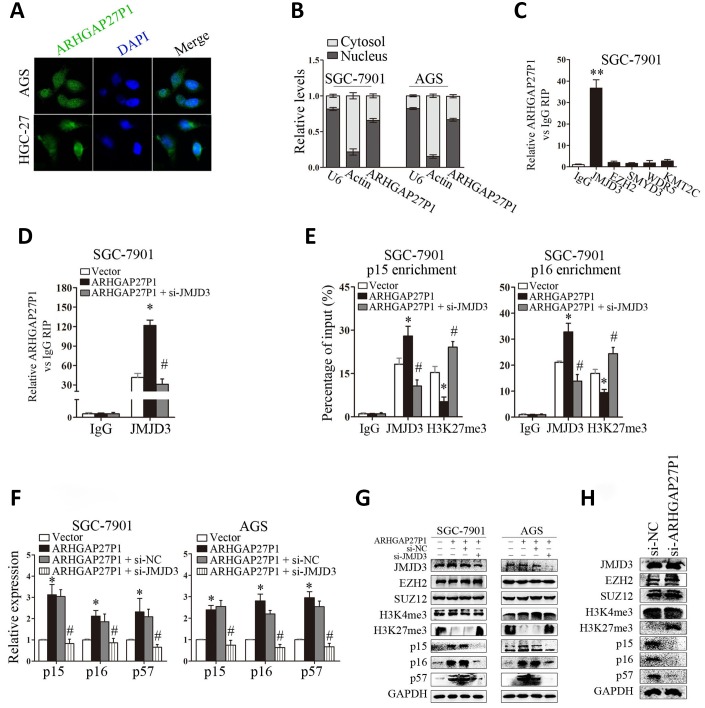
**ARHGAP27P1 epigenetically activated p15 and p16 transcription by binding to JMJD3.** (**A**) FISH analysis of the subcellular location of ARHGAP27P1 in AGS and HGC-27 cells. (**B**) Subcellular fractionation and RT-qPCR analysis to determine the ARHGAP27P1 location. U6 and β-actin were used as nucleus and cytoplasm markers, respectively. (**C**) RIP experiments were performed in SGC-7901 cells with IgG, anti-JMJD3, EZH2, SMYD3, WDR5 or KMT2C antibody, and the coprecipitated RNA was subjected to RT-qPCR analysis for ARHGAP27P1. ARHGAP27P1 expression levels were presented as fold enrichment in diverse immunoprecipitates relative to that of IgG. (**D**) RIP assays were performed to determine the binding of ARHGAP27P1 to JMJD3 in SGC-7901 cells transfected with pcDNA-ARHGAP27P1, or cotransfected with pcDNA-ARHGAP27P1 and si-JMJD3. (**E**) ChIP-qPCR of JMJD3 occupancy and H3K27me3 binding to the p15 and p16 promoters in SGC-7901 cells, IgG as a negative control. (**F**) ARHGAP27P1-overexpressing SGC-7901 or AGS cells were cotransfected with si-JMJD3. The expression of p15, p16 and p57 was determined using RT-qPCR. (**G**) The protein levels of JMJD3, EZH2, SUZ12, H3K4me3, H3K27me3, p15, p16 and p57 were determined by Western blot. (**H**) Western blot analysis of JMJD3, EZH2, SUZ12, H3K4me3, H3K27me3, p15, p16 and p57 in HGC-27 cells transfected with si-ARHGAP27P1. *P < 0.05, **P < 0.01 versus vector or control. #*P* < 0.05 versus ARHGAP27P1.

Although we showed that ARHGAP27P1 affected the expression of p15, p16 and p57, the underlying mechanisms were still unclear. To address this issue, we firstly examined the subcellular localization of ARHGAP27P1 by fractionation assay and FISH assay. The results showed that ARHGAP27P1 mainly resided in the nucleus of GC cells (Figure 5A and 5B), suggesting that ARHGAP27P1 might play a major regulatory function at transcriptional level. Recent studies have reported that an increasing number of lncRNAs function in cooperation with chromatin modifying enzymes to promote epigenetic activation or silencing of gene expression [13]. The association of lncRNAs with PRC2 is among the best characterized models for this interaction [14, 15, 29]. It has been shown that approximately 20% of all human lncRNAs physically associate with PRC2, suggesting that lncRNAs may have a general role in recruiting PRC2 to their target genes [14]. Other epigenetic modifiers including LSD1, SMYD3, WDR5, and KMT2C may also function as potential partners for lncRNA [16–18]. Therefore, we screened a panel of chromatin modifiers (JMJD3, EZH2, SMYD3, WDR5, KMT2C) that could potentially interact with lncRNAs by RIP experiments in SGC-7901 cells. The results showed that endogenous ARHGAP27P1 was specifically enriched in the anti-JMJD3 RIP fraction relative to the input compared to the IgG fraction (Figure 5C). These results suggested that ARHGAP27P1 could specifically bind to JMJD3 rather than other chromatin modifiers. Moreover, overexpression of ARHGAP27P1 enhanced the interaction of ARHGAP27P1 with JMJD3, an effect further reversed by silencing of JMJD3 (Figure 5D). JMJD3 (also named KDM6B) is a family member of the histone H3K27me3-specific demethylases that promote gene transcription mainly by acting as the rivals of PRC2 that otherwise catalytically adds the methyl groups to H3K27 [30, 31]. Here, we observed that enhanced interaction of ARHGAP27P1 with JMJD3 by ARHGAP27P1 overexpression led to remarkable demethylation of H3K27me3 in SGC-7901 and AGS cells, while ARHGAP27P1 silencing in HGC-27 cells showed the opposite effect (Figure 5G and 5H). JMJD3 is well documented to mediate the oncogenic stress-induced cell senescence via activating INK4 gene locus (INK4b-ARF-INK4a) and/or facilitating the regulatory activity of p53 on its target genes [32–34], suggesting an oncorepressor activity of JMJD3 in human malignancies via cell senescence induction. We then asked whether the interaction of ARHGAP27P1 with JMJD3 was required for ARHGAP27P1-mediated transcriptional activation of the CKIs. As expected, we observed that knockdown of JMJD3 reversed ARHGAP27P1-mediated H3K27me3 demethylation, thereby inhibiting p15, p16 and p57 expression in SGC-7901 and AGS cells (Figure 5F and 5G). We further showed that the levels of JMJD3, EZH2, SUZ12 and H3K4me3 were not affected by ARHGAP27P1 overexpression or knockdown. Consistently, the mRNA or protein levels of JMJD3 were not associated with ARHGAP27P1 expression levels in the detected cancerous tissues (Figure 4F and 4G). However, high expression levels of ARHGAP27P1 in cancerous tissues were associated with low levels of H3K27me3, suggesting that the interaction of ARHGAP27P1 with JMJD3 was crucial for H3K27me3 demethylation and transcriptional activation of p15, p16, and p57 (Figure 4G). Collectively, these results indicated that the association of ARHGAP27P1 with JMJD3 as well as the demethylation of H3K27me3 were specifically required for ARHGAP27P1-mediated activation of p15, p16 and p57.

Next, we sought to determine whether ARHGAP27P1 was involved in transcriptional activation through enrichment of JMJD3 to target gene promoters. ChIP assays showed that JMJD3 could bind to the p15 and p16 promoter regions (Figure 5E). Overexpression of ARHGAP27P1 enhanced JMJD3 binding to the p15 and p16 promoter regions, an effect further reversed by knockdown of JMJD3 (Figure 5E). Consistently, knockdown of JMJD3 counteracted the reduction in H3K27me3 binding to the p15 and p16 promoter regions by ARHGAP27P1 overexpression (Figure 5E). Taken together, these results suggested that ARHGAP27P1 acted to target JMJD3 occupancy and activity to epigenetically modulate the expression of p15, p16 and p57.

### Silencing of JMJD3, p15 or p16 reversed the tumor suppressive effect of ARHGAP27P1

**Figure 6:**
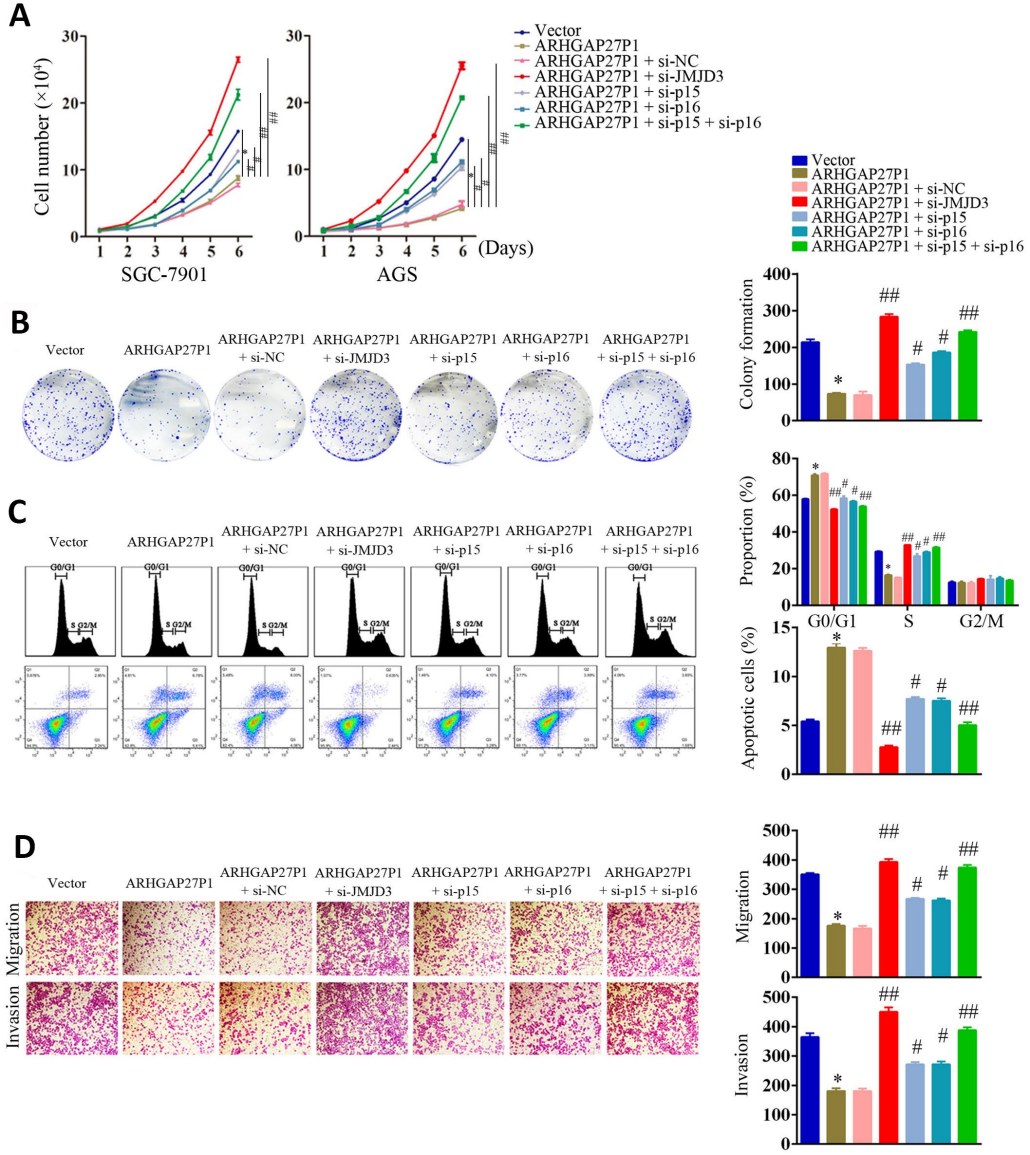
**Silencing of JMJD3, p15 or p16 reversed the suppressive effect of ARHGAP27P1 in GC malignant progression.** (**A**) ARHGAP27P1-overexpressing SGC-7901 and AGS cells were cotransfected with si-JMJD3, si-p15 or si-p16. Cell proliferation was determined by cell counting assays. (**B**) Colony formation assays. (**C**) Cell cycle assays and cell apoptosis assays. (**D**) Transwell migration and invasion assays. *P < 0.05 versus vector. #*P* < 0.05, ##*P* < 0.01 versus ARHGAP27P1.

To further validate that JMJD3, p15 and p16 were involved in ARHGAP27P1-mediated phenotype changes in GC cells, we performed rescue experiments. As shown in Figure 6A–6D and Supplementary Figure 2A–2C, silencing of p15 or p16 in SGC-7901 and AGS cells partially reversed the inhibitory effects against proliferation, cell cycle progression, migration and invasion induced by ARHGAP27P1 overexpression. Double knockdown of p15 and p16 completely reversed the above effects by ARHGAP27P1 overexpression. Consistently, silencing of JMJD3 showed the comparable effects as double knockdown of p15 and p16. Collectively, these results indicated that ARHGAP27P1 inhibited proliferation, cell cycle progression, migration and invasion of GC cells through JMJD3/p15/p16 signaling pathway.

### Overexpression of ARHGAP27P1 inhibited GC tumorigenesis *in vivo*

**Figure 7:**
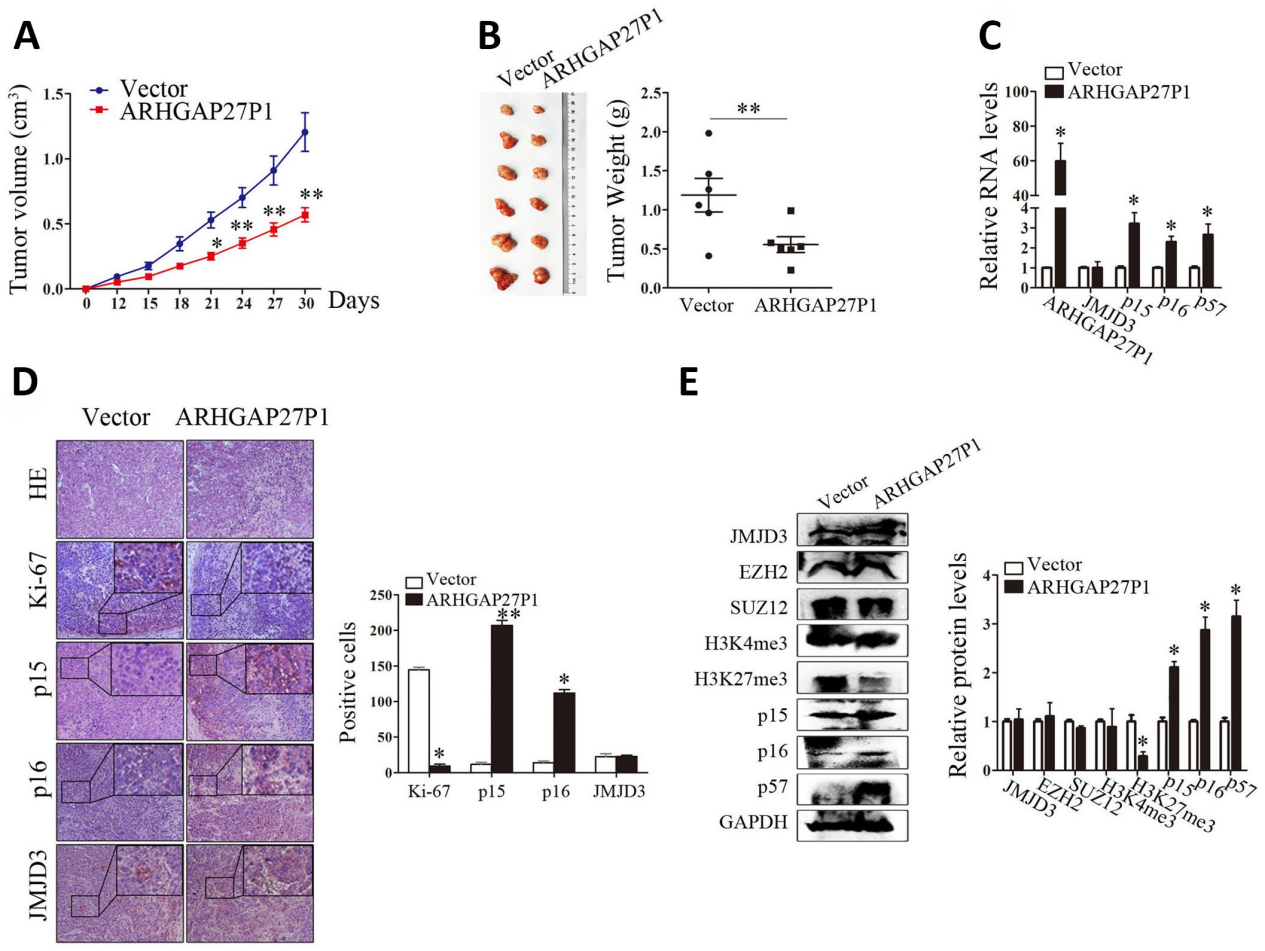
**Overexpression of ARHGAP27P1 inhibited tumor growth *in vivo***. (**A**) Growth curves of tumors from vector or ARHGAP27P1 groups were shown. Tumor volumes were calculated every 3 days. (**B**) Tumor samples and tumor weights from respective groups were represented. (**C**) RT-qPCR analysis of ARHGAP27P1, JMJD3, p15, p16 and p57 in xenograft tumors. (**D**) Histopathology and immunostainings of JMJD3, p15, p16 and Ki-67 in paraffin tumor sections. (**E**) Representative blots of JMJD3, EZH2, SUZ12, H3K4me3, H3K27me3, p15, p16, and p57 in tumor tissues. *P < 0.05, **P < 0.01 versus vector.

To determine whether ARHGAP27P1 affected tumor formation *in vivo*, we injected SGC-7901 cells stably transfected with ARHGAP27P1 or empty vector into nude mice. In consistent with *in vitro* results, tumor growth in the ARHGAP27P1 group was obviously slower than that in the vector group (Figure 7A). Up to 30 days after injection, the tumor weight in the ARHGAP27P1 group was significantly lower than that in the vector group (Figure 7B). Next, RT-qPCR assays confirmed high ARHGAP27P1 expression in tumor tissues collected from ARHGAP27P1 group rather than vector group (Figure 7C). Accordingly, the ARHGAP27P1 group associated with higher mRNA and protein expression levels of p15, p16, and p57 (Figure 7C–7E). This might be explained by decreased H3K27me3 levels following ARHGAP27P1 overexpression (Figure 7E). In addition, the expression of JMJD3, EZH2, SUZ12, or H3K4me3 was not affected by ARHGAP27P1 overexpression (Figure 7E). We also found that tumors developed from ARHGAP27P1-overexpressing cells showed less Ki-67 expression than tumors from vector group (Figure 7D). These data further supported a tumor suppressor role of ARHGAP27P1 in GC development.

## DISCUSSION

In this study, we for the first time demonstrated a tumor suppressor role of ARHGAP27P1 in GC tumorigenesis. The ARHGAP27P1 expression was dramatically downregulated in GC tissues, and its low expression was closely related to advanced TNM stage, increased invasion depth and lymphatic metastasis. Low ARHGAP27P1 expression also independently predicted a poor OS of GC patients. In addition, circulating ARHGAP27P1 levels showed a moderate accuracy for diagnosis of GC. The tumorigenesis of GC is a very complicated pathological process, which involves malignant proliferation, abnormal apoptosis, migration and invasion of GC cells. Gain- and loss-of-function of ARHGAP27P1 assays revealed that ARHGAP27P1 inhibited GC cell proliferation, cell cycle progression, invasion, and migration and induced apoptosis. Therefore, ARHGAP27P1 might be explored as a potential diagnostic indicator and therapeutic target for GC. Nevertheless, given that the expressional differences of ARHGAP27P1 between cancerous tissues/patients and noncancerous tissues/patients were not large, and that overexpression of ARHGAP27P1 did not completely abolish GC growth *in vitro*/*in vivo*, these finding were still worth further investigation.

As a novel documented lncRNA, the possible mechanisms by which ARHGAP27P1 regulated the biological phenotypes of GC were currently unclear. We screened the key genes associated with GC occurrence and progression and found that ARHGAP27P1 mainly affected the expression of p15, p16 and p57, but not the other candidate targets. P15, p16 and p57, which are major members of CKIs, bind directly to G1/S-transformed kinases (such as cyclin D/cyclin-dependent kinase (CDK)-4 and cyclin D/CDK6) to regulate a variety of cellular biological processes [35, 36]. Specifically, CDKs and their cyclin partners are positive regulators or accelerators that induce cell cycle progression; whereas, CKIs that act as brakes to stop cell cycle progression in response to regulatory signals are important negative regulators [37]. Transcription of CKIs is a key requirement for replicative or oncogene-induced senescence and constitutes an important barrier for tumor growth, while inactivation of CKIs has long been demonstrated to result in cell cycle disorders and boost cell proliferation in human cancers [38].

Our subcellular localization experiments identified a nuclear enrichment of ARHGAP27P1, suggesting that ARHGAP27P1 might play a major regulatory function at transcriptional level. Indeed, epigenetic modifiers are among the most frequent protein partners of lncRNAs that have been identified to date, of which histone methyltransferases and protein members of the PRC2 have received considerable attention [14, 29, 31, 38]. It has been reported that approximately 20% of all human lncRNAs act to physically associate with PRC2, suggesting that lncRNAs may have a general role in recruiting PRC2 to their target genes [14]. Other epigenetic modifiers including LSD1, SMYD3, WDR5, KMT2C, SET1C, ING2 and PAQR3 may also function as potential candidates for lncRNA partners [16–18]. We screened a panel of chromatin modifiers (JMJD3, EZH2, SMYD3, WDR5, KMT2C) by RIP experiments and found that endogenous ARHGAP27P1 was enriched in the anti-JMJD3 RIP fraction compared to the IgG fraction. These results suggested that ARHGAP27P1 could specifically bind to JMJD3 rather than other chromatin modifiers. To the best of our knowledge, the role of JMJD3 as a lncRNA partner has not been reported before. Actually, previous studies regarding the expression and the role of JMJD3 in different cancers have shown discrepancies [30, 32, 33, 39–46]. For example, JMJD3 is well documented to mediate the oncogenic stress-induced cell senescence via activating INK4 gene locus, suggesting an oncorepressor activity of JMJD3 in human malignancies [30, 32, 33]. JMJD3 has also been shown to exert anti-acute myeloid leukemia (AML) effect by directly modulating H3K4 and H3K27 methylation levels to activate the expression of a number of key myelopoietic regulatory genes [39]. Consistently, JMJD3 deficiency was reported to promote malignant progression of human pancreatic carcinoma by decreasing the expression of C/EBPα [40], a potential inhibitory partner of E2F1. Moreover, JMJD3 expression levels are lower in various cancers, including CRC and liver carcinoma [43, 44]. On the other hand, an oncogenic role of JMJD3 has been documented in the case of T-cell acute lymphocytic leukemia (T-ALL) and myelodysplastic syndrome (MDS) [45, 46], which was largely attributed to its specific partnership with NF-κB and NOTCH, two transcription factors whose overactivations are highly associated with the induction of multiple types of inflammatory cytokines and the proliferation of T cells. JMJD3 has also been recently shown upregulated in GC and ESCC, and its high expression predicted unfavorable survival [41, 42]. These observations implicate the existence of uncharacterized regulatory effects of JMJD3 on cell differentiation, survival, and proliferation, and suggest that the expression patterns of JMJD3 are context and cancer type specific. Given that the downstream targets of JMJD3 are huge in quantity and diverse in their functions, to investigate the specific downstream signaling activated by JMJD3 should be of vital importance according to different contexts. Apart from the expression levels of JMJD3, we argue that the interaction of JMJD3 with its different partners should also be taken into account to have a better understanding of this issue. Indeed, our results showed that overexpression of ARHGAP27P1 enhanced the interaction of ARHGAP27P1 with JMJD3, but not the expression levels of JMJD3. We further found that the increase in association of ARHGAP27P1 with JMJD3 led to remarkable decrease in H3K27me3 levels, thereby promoting p15, p16 and p57 transcription. Consistently, knockdown of JMJD3 reversed the above effects due to decrease in the interaction of ARHGAP27P1 with JMJD3. In addition, ChIP assays suggested that ARHGAP27P1 acted to target JMJD3 occupancy and activity to epigenetically modulate the expression of p15, p16 and p57. Therefore, it was not surprising that JMJD3, p15 and p16 were crucially involved in ARHGAP27P1-mediated phenotype changes of GC cells.

Taken together, our study identified a novel ARHGAP27P1-mediated regulator of the GC cell cycle and proliferation. ARHGAP27P1 serving as a member of JMJD3-mediated epigenetic regulation participated in the occurrence and development of GC. Our study may expand our understanding of the role of lncRNAs as an epigenetic regulator in GC, and provide a strategy and facilitate the development of lncRNA-directed diagnostics and therapeutics against this disease.

## MATERIALS AND METHODS

### Patients and specimens

Human GC specimens were obtained from the Affiliated People’s Hospital of Jiangsu University. This study was approved by the Institutional Ethical Committee of Jiangsu University, and informed consents were signed by all patients. Corresponding normal gastric tissue samples were taken from tissues that were located 5 cm away from tumor margin. All selected GC patients met the following inclusion criteria: (1) Patients were newly diagnosed to have GC with definite pathological evidence or radiological evidence; (2) No chemoradiotherapies were given before surgery. Plasma samples were also collected from each subject and were stored at −80°C before use. Plasma ARHGAP27P1 expression was detected in 53 randomly selected GC patients and 53 age and gender well matched healthy donors. Tumor stage was evaluated according to the eighth TNM staging (T for characteristics of the primary tumor, N for nodal involvement, and M for distant metastasis) of the International Union against Cancer (UICC)/American Joint Committee on Cancer (AJCC) system [47].

### Cell culture and transfection

Five human GC cell lines SGC-7901, MGC-803, HGC-27, AGS and BGC-823 were obtained from the Cell Bank of the Chinese Academy of Sciences (Shanghai, China). The human normal gastric epithelial cell line (GES-1) was purchased from Procell Life Science and Technology (Wuhan, China). Cells were cultured in Dulbecco’s modified Eagle’s medium (DMEM; Thermo Fisher Scientific, USA) or Roswell Park Memorial Institute (RPMI) 1640 medium (Thermo Fisher Scientific, USA). All media were supplemented with 10% fetal bovine serum (FBS; Gibco, USA), 100 U/ml penicillin and 100μg/ml streptomycin (Gibco, USA) in humidified air at 37°C with 5% CO_2_.

### RNA isolation and reverse transcription-quantitative polymerase chain reaction (RT-qPCR)

Total RNA was extracted from tissues, plasma and cells of GC using Trizol Reagent (Sangon Biotech, Shanghai, China). For RT-qPCR, RNA was reversely transcribed into complementary DNA (cDNA) by using a Hieff First Strand cDNA Synthesis Super Mix for RT-qPCR kit (Yeasen Biotech, Shanghai, China). Quantitative PCR analysis was carried out using the Hieff qPCR SYBR Green Master Mix kit (Yeasen Biotech, Shanghai, China) on ABI 7500 real-time PCR system (Applied Biosystems, Singapore). The primer sequences were shown in Supplementary Table 1. β-actin or U6 was used as an endogenous control depending on the gene/sample detected. Relative expression levels were calculated by using the 2^−ΔΔCt^ method.

### Constructs, synthesized oligos and treatment

The ARHGAP27P1 overexpression plasmid was purchased from GenePharm (Shanghai, China). Briefly, the ARHGAP27P1 sequence was synthesized and inserted into the pcDNA3.1 (+) vector at the BamH1 sites. The small interfering RNAs (siRNAs) for ARHGAP27P1, JMJD3, p15 and p16, as well as scrambled negative control oligos, were also synthesized by GenePharm. The siRNA sequences are listed as follows: si-ARHGAP27P1 sense: 5′-GAGGAACGCUU GACAAAGUTT-3′, antisense: 5′-ACUUUGUCAAGC GUUCCUCTT-3′; si-JMJD3 sense: 5′-GUGACAAGGA GAGACCUUUAUTT-3′, antisense: 5′-AUAAAGGUC UCCUUGUCACTT-3′; si-p15 sense: 5′-CCAACGGAG UCAACCGUUUTT-3′, antisense: 5′-AAACGGUUGA CUCCGUUGGTT-3′; si-p16 sense: 5′-CCCAACGCAC CGAAUAGUUTT-3′, antisense: 5′-AACUAUUCGGU GCGUUGGGTT-3′; si-NC sense: 5′-UUCUCCGAACG UGUCACGUTT-3′, antisense: 5′-ACGUGACACGUU CGGAGAATT-3′. For transfection, the cells were grown in a 12-well plate until confluence at 60%-80% and were transfected with the indicated molecules with Lipofectamine 2000 (Thermo Fisher Scientific), according to the manufacturer’s instructions. Forty-eight hours after transfection, the cells were ready for the following experiments.

### Cell counting and colony formation assay

For proliferation assay, GC cells were seeded into 24-well plates (1 × 10^4^ cells/well). The cells were collected and counted every day for 6 days. The results were plotted as cell growth curves. For colony formation assay, GC cells were placed in 6-well plates (1000 cells/well) and incubated at 37°C in a 5% CO_2_ humidified incubator. The culture medium was changed every 3 days. After 10 days, the colonies were washed with phosphate buffered saline (PBS), fixed with 4% paraformaldehyde for 10 minutes and stained with 0.5% crystal violet for 5 minutes. Visible colonies were counted and photographed. Experiments were carried out in triplicate independently.

### Cell apoptosis and cell cycle analysis

For cell apoptosis assay, the indicated cells were stained with Annexin V-FITC/PI cell apoptosis detection kit (BD Pharmingen, San Diego, CA) and analyzed with a flow cytometer (BD FACSCalibur, San Jose, CA). Cells for cell cycle analysis were stained with PI using the CycleTEST PLUS DNA Reagent kit (BD Biosciences, Franklin Lakes, NJ, USA) according to the protocol. The percentages of cells in G0/G1, S and G2/M phase were counted and compared.

### Migration and invasion assay

Migration and invasion assays were performed in transwell chambers without or with Matrigel-coated membranes, respectively. Briefly, GC cells were seeded with serum-free medium into the upper chambers at 5 × 10^4^ cells/well, and the bottom chambers contained medium with 10% FBS. After incubation at 37°C with 5% CO_2_ for 24 hours, cells on the upper surface were removed with a cotton swab and cells on the lower surface were fixed with 4% paraformaldehyde for 10 minutes and stained with 0.5% crystal violet for 5 minutes. Cells were photographed and counted under an inverted microscope (Nikon, Japan).

### Wound-healing assays

Indicated cells were plated to confluence in 6-well plates. Streaks across the plate were created in the monolayer with a pipette tip, followed by three washes with PBS. The cells were then cultured for hours with medium containing 1% FBS. To ensure documentation of the same region, the wells were marked across the wounded area. Progression of migration was observed and photographed at 0 and 24 hours after wounding. The distance between the two edges of the scratch was measured and calculated.

### Subcellular fractionation

The separation of cytoplasmic and nuclear fractions was performed using the PARIS Kit (Life Technologies, Carlsbad, CA, USA) according to the manufacturer’s instructions.

### Fluorescence In Situ Hybridization (FISH)

The ARHGAP27P1 FISH probes (probe 1#: 5′-GCTTGCCGAGGCTGAGGGACCGAA-3′, Probe 2#: 5′-TGTCCGTGCGGAGTCCCAAAGCAGG-3′) were designed and synthesized by GenePharm (Shanghai, China). Briefly, cells were washed and fixed with 4% paraformaldehyde for 10 min. After treatment with protease K (2 μg/mL), glycine and phthalide reagent, the cells were incubated with 100 μL of pre-hybridization solution at 42°C for 1 h. After that, the cells were hybridized with 100 μl of hybridization solution containing probes (10 μM) at 73°C for 5 min and 37°C for 12–16 h in a humidified incubator. Then, the cells were counterstained with 4′,6-diamidino-2-phenylindole (DAPI) and observed under a fluorescence microscope (Leica, SP8 laser confocal microscope).

### Western blot

Equal amounts of protein samples were separated by 8%-15% sodium dodecyl sulfate-polyacrylamide gel electrophoresis (SDS-PAGE) and were transferred to nitrocellulose membranes. Bands were probed immunologically using antibodies against JMJD3, EZH2, SUZ12, H3K4me3, H3K27me3, p15, p16, p57 and GAPDH. Antibodies against JMJD3, EZH2, SUZ12, H3K4me3, H3K27me3 were purchased from Cell signaling Technology (MA, USA). Antibodies against p15, p16, p57 and GAPDH were purchased from EnoGene (Nanjing, China). Signals were detected using an enhanced chemiluminescence (ECL) system according to the manufacturer’s instructions.

### Immunohistochemistry

Briefly, sections were deparaffinized, rehydrated, and incubated in sodium citrate buffer for antigen retrieval. Endogenous peroxidase was blocked by incubation in 3% H_2_O_2_ for 10 min at room temperature. The sections were then incubated with antibodies against p15, p16, JMJD3 or Ki-67 (Santa Cruz, CA) overnight at 4°C. Finally, HRP-conjugated secondary antibody and diaminobenzidine (DAB) solution (Solarbio, Beijing, China) were used to detect the signals. Slides were photographed under a microscope.

### Chromatin immunoprecipitation (ChIP) assays

ChIP experiment was conducted using the EZ-CHIP KIT (Millipore, Billerica, MA, USA). Briefly, the DNA-protein crosslink was built through GC cells’ incubation with formaldehyde. Cell lysates were then sonicated to generate chromatin fragments of 200-300 bps and immunoprecipitated with anti-JMJD3 or H3K27me3 antibodies. Precipitated chromatin DNA was recovered and analyzed by qPCR. The ChIP primer sequences for p15 and p16 are listed in Supplementary Table 1. The results were calculated as a percentage relative to the input DNA.

### RNA immunoprecipitation (RIP)

RIP experiments were performed using a Magna RIP RNA-Binding Protein Immunoprecipitation Kit (Millipore, USA) according to the manufacturer’s instructions. Antibodies for JMJD3, EZH2, SMYD3, WDR5 and KMT2C RIP assays were from Cell signaling (MA, USA).

### Tumor xenograft experiment

The procedures for animal studies were approved by the Animal Use and Care Committee of Jiangsu University. Four-week-old athymic BALB/c mice were maintained under specific pathogen-free conditions. Briefly, SGC-7901 cells stably transfected with vector or ARHGAP27P1 were selected by G418 (Invitrogen, Carlsbad, CA). Approximately 2 × 10^6^ SGC-7901 cells were resuspended with 0.2 ml PBS and injected into the dorsal right flank of each nude mouse (6 mice per group). Tumor volumes were calculated as 0.5 × length × width^2^ every 3 days. After 30 days, the mice were sacrificed, and tumors were excised, weighed, and immediately frozen at -80°C for future use.

### Statistical analysis

All statistical analyses were conducted with SPSS 24.0 software (IBM, SPSS, USA). The significance of differences between groups was estimated by Student’s t-tests or one-way analysis of variance (ANOVA). The associations between ARHGAP27P1 and the clinicopathological features were analyzed by the Pearson χ^2^ test. The log-rank test was used to identify statistically significant differences between survival curves. Univariate and multivariate Cox proportional hazards analyses were carried out to evaluate the independent risk factors for the OS of patients. Receiver operating characteristic (ROC) curve analysis was used to investigate the predictive value of ARHGAP27P1 in differentiating patients with GC from the noncancerous population. The area under the ROC curve (AUC) indicated the accuracy of diagnosis. A *P* values less than 0.05 was considered statistically significant.

## Supplementary Material

Supplementary Figures

Supplementary Table 1
