# Supplementary material for: Long noncoding RNA ARHGAP27P1 inhibits gastric cancer cell proliferation and cell cycle progression through epigenetically regulating p15 and p16
Source: Aging (Albany NY). 2019 Oct 30;11(20):9090–110. doi: 10.18632/aging.102377 (PMC6834409; doi:10.18632/aging.102377)
Supplement: Supplementary Figures [file aging-11-102377-s002.pdf]

SUPPLEMENTARY FIGURES

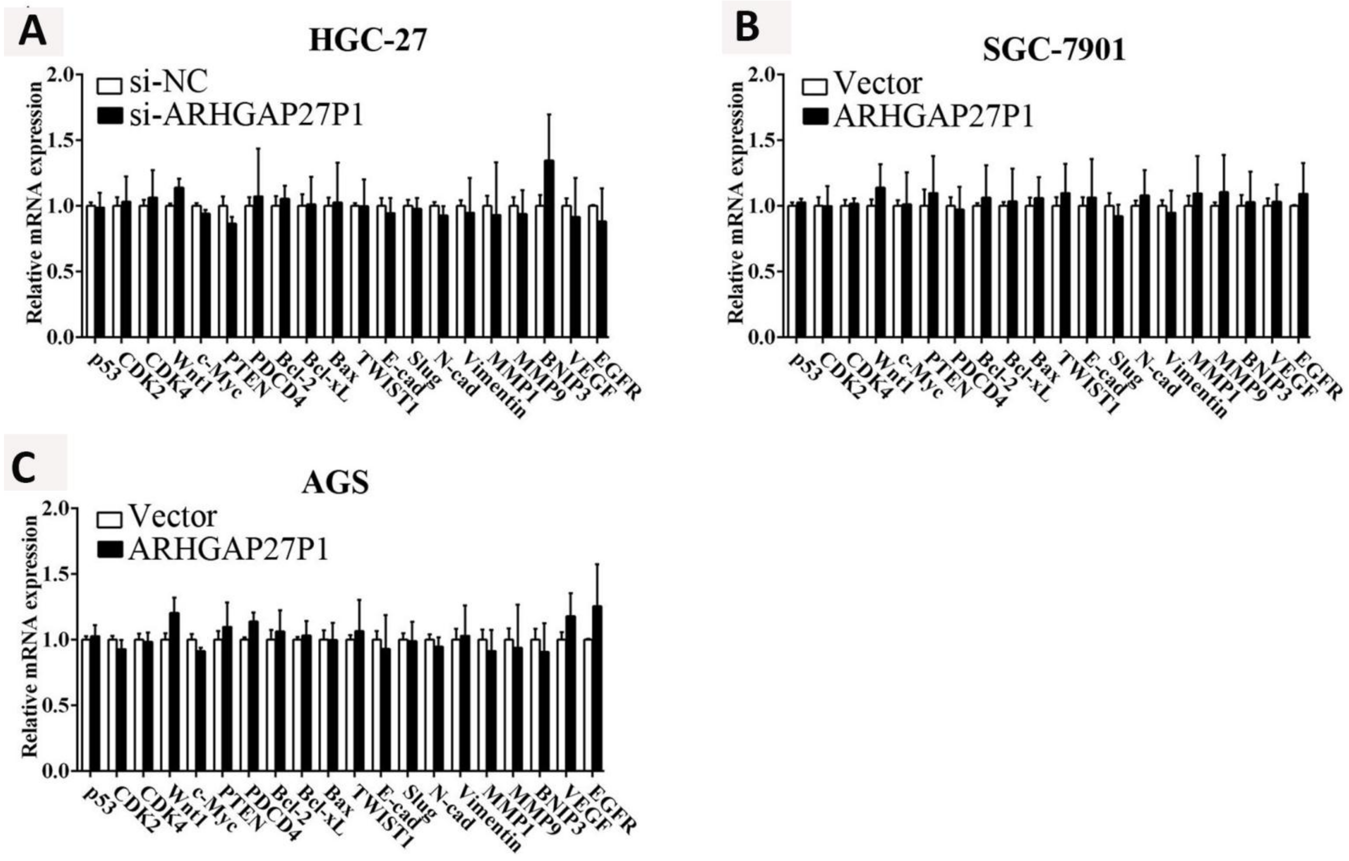

Supplementary Figure 1. ARHGAP27P1 showed minimal effects on the expression of diverse potential genes involved in GC progression. (**A**) RT-qPCR analysis of p53, CDK2, CDK4, Wnt1, c-Myc, PTEN, PDCD4, Bcl-2, Bcl-xL, Bax, TWIST1, E-cad, Slug, N-cad, Vimentin, MMP1, MMP9, BNIP3, VEGF and EGFR in HGC-27 cells transfected with si-ARHGAP27P1 or si-NC. (**B** and **C**) RT-qPCR analysis of the above genes in SGC-7901 or AGS transfected with pcDNA-ARHGAP27P1 or pcDNA.

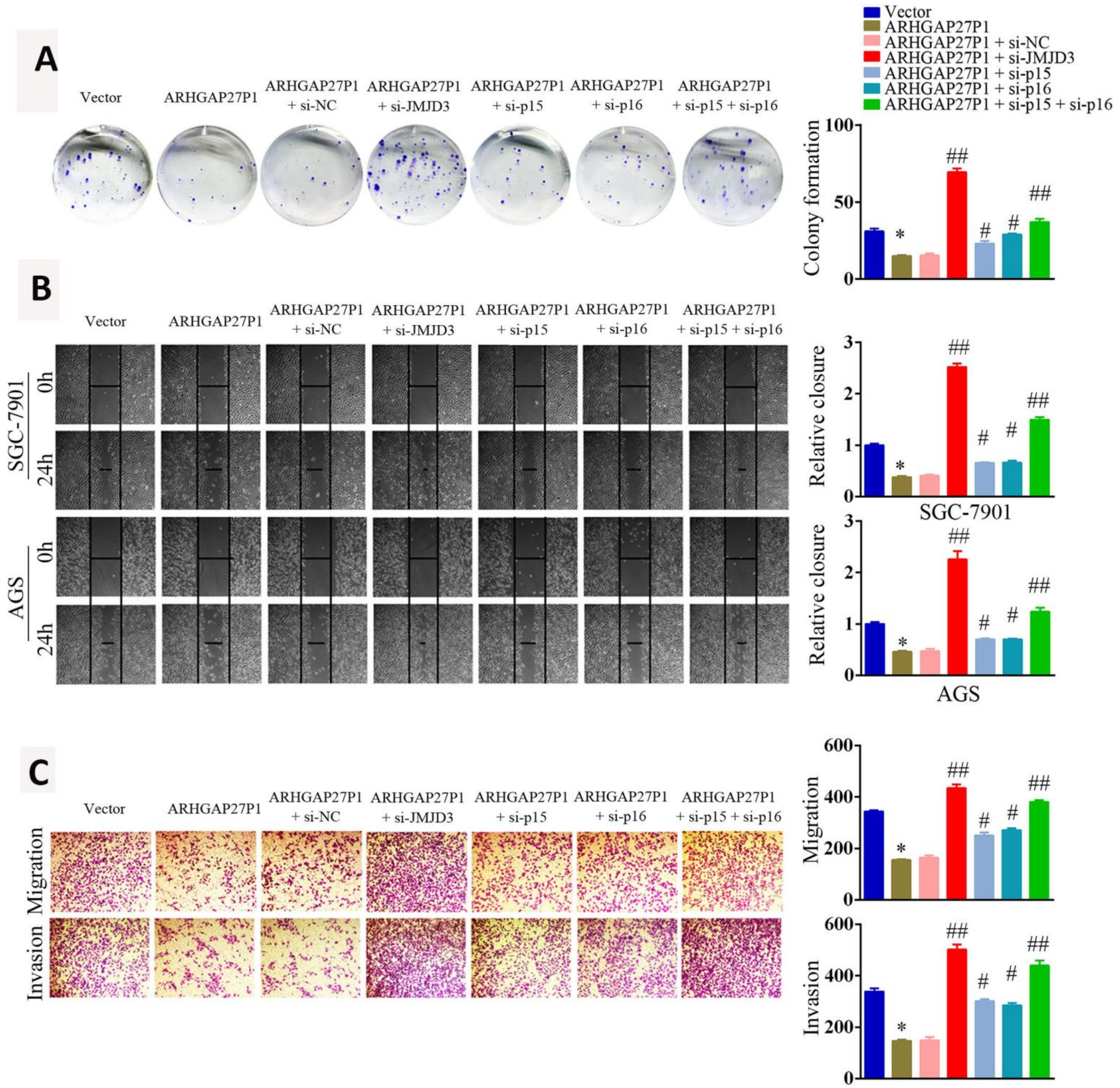

Supplementary Figure 2. Silencing of JMJD3, p15 or p16 counteracted the tumor suppressive effect of ARHGAP27P. (**A**) ARHGAP27P1-overexpressing AGS cells were cotransfected with si-JMJD3, si-p15 or si-p16. Cell proliferation was determined by colony formation assays. (**B**) Wound-healing assays for ARHGAP27P1-overexpressing SGC-7901 or AGS cells cotransfected with si-JMJD3, si-p15 or si-p16. (**C**) Migration and invasion assays for ARHGAP27P1-overexpressing AGS cells cotransfected with si-JMJD3, si-p15 or si-p16. *$P < 0.05$ versus vector. #$P < 0.05$, ##$P < 0.01$ versus ARHGAP27P1.
