# Supplementary material for: Long noncoding RNA ARHGAP27P1 inhibits gastric cancer cell proliferation and cell cycle progression through epigenetically regulating p15 and p16
Source: Aging (Albany NY). 2019 Oct 30;11(20):9090–110. doi: 10.18632/aging.102377 (PMC6834409; doi:10.18632/aging.102377)
Supplement: Supplementary Table 1 [file aging-11-102377-s001.pdf]

SUPPLEMENTARY TABLE

Supplementary Table 1. Gene primer sequences.

Gene name	Forward(5′-3′)	Reverse(5′-3′)
ARHGAP27P1	CGGGTCCCCTTCTTATCCAG	ATTGGCACCCTGAATCACCC
p15	GGGAGGGTAATGAAGCTGAG	GGCCGTAAACTTAACGACACT
p16	CTACTGAGGAGCCAGCGTCT	CTGCCCATCATCATGACCT
p21	TGTGGACCTGTCACTGTCTTGTA	GGCGTTTGGAGTGGTAGAAATCT
p27	GGCAAGTACGAGTGGCAAGAG	AATGCGTGTCCTCAGAGTTAGC
p57	TGAACGCCGAGGACCAGAA	ACCGAGTCGCTGTCCACTT
p53	GCTCTGACTGTACCACCATCC	CTCTCGGAACATCTCGAAGCG
CDK2	GTACCTCCCCTGGATGAAGAT	CGAAATCCGCTTGTTAGGGTC
CDK4	CTGGTGTTTGAGCATGTAGACC	GATCCTTGATCGTTTCGGCTG
Wnt1	ATCTTCGCTATCACCTCCGC	GGCCGAAGTCAATGTTGTCG
c-Myc	GGACTTGTTGCGGAAACGAC	CTCAGCCAAGGTTGTGAGGT
PTEN	GCACTGTTGTTTCACAAGATGATG	GCAGACCACAAACTGAGGATTG
Bcl-2	CTGGGATGCCTTTGTGGAAC	CAGGCATGTTGACTTCACTTGT
PDCD4	GCTACCGTGCTTCTGAGTAT	GGCAATGTTCAGCTTCAGAT
Bax	GTCGCCCTTTTCTACTTTGCC	AGTCGCTTCAGTGACTCGG
Bcl-xL	CCCAGAAAGGATACAGCTGG	GCGATCCGACTCACCAATAC
TWIST1	GGAGTCCGCAGTCTTACGAG	TCTGGAGGACCTGGTAGAGG
E-cad	GGTCTGTCATGGAAGGTGCTC	CAGGATCTTGGCTGAGGATGG
Slug	TGTGACAAGGAATATGTGAGCC	TGAGCCCTCAGATTTGACCTG
N-cad	TCAACTGCAACCGTGTCTGT	ATCGATCTGGGTCCTGAGCA
MMP1	CACAAACCCCAAAAGCGTGT	TCGGCAAATTCGTAAGCAGC
MMP9	CAAGTGGCACCACCACAACATCA	CCCGCGGCAAGTCTTCCGA
Vimentin	TGGACCAGCTAACCAACGAC	GCCAGAGACGCATTGTCAAC
BNIP3	TTTAAACACCCGAAGCGCAC	CTGGTGGAGGTTGTCAGACG
VEGF	GAGATGAGCTTCCTACAGCACA	TCACCGCCTCGGCTTGTC
EGFR	CCTGGTCTGGAAGTACGCAG	CGATGGACGGGATCTTAGGC
JMJD3	ACACCTTGAGCACAAACGGA	TTCAGAGTTGCAGCCTCTCC
β-actin	CATTCCAAATATGAGATGCGTTGT	TGTGGACTTGGGAGAGGACT
U6	CTCGCTTCGGCAGCACA	AACGCTTCACGAATTTGCGT
p15-ChIP	TCCAGTTAAGCCTACCATGACAG	AACTCCTCTGTGGCATGTGTC
p16-ChIP	AAGAAGAAGCCATACTTTCCCTATG	GCGTGTTTGAGTGCGTTCA
